# Neurotelemetry Reveals Putative Predictive Activity in HVC during Call-Based Vocal Communications in Zebra Finches

**DOI:** 10.1523/JNEUROSCI.2664-19.2020

**Published:** 2020-08-05

**Authors:** Shouwen Ma, Andries ter Maat, Manfred Gahr

**Affiliations:** Department of Behavioural Neurobiology, Max Planck Institute for Ornithology, 82319, Seewiesen, Germany

**Keywords:** sensorimotor, prediction, vocal communication, zebra finch

## Abstract

Premotor predictions facilitate vocal interactions. Here, we study such mechanisms in the forebrain nucleus HVC (proper name), a cortex-like sensorimotor area of songbirds, otherwise known for being essential for singing in zebra finches. To study the role of the HVC in calling interactions between male and female mates, we used wireless telemetric systems for simultaneous measurement of neuronal activity of male zebra finches and vocalizations of males and females that freely interact with each other. In a non-social context, male HVC neurons displayed stereotypic premotor activity in relation to active calling and showed auditory-evoked activity to hearing of played-back female calls. In a social context, HVC neurons displayed auditory-evoked activity to hearing of female calls only if that neuron showed activity preceding the upcoming female calls. We hypothesize that this activity preceding the auditory-evoked activity in the male HVC represents a neural correlate of behavioral anticipation, predictive activity that helps to coordinate vocal communication between social partners.

**SIGNIFICANCE STATEMENT** Most social-living vertebrates produce large numbers of calls per day, and the calls have prominent roles in social interactions. Here, we show neuronal mechanisms that are active during call-based vocal communication of zebra finches, a highly social songbird species. HVC, a forebrain nucleus known for its importance in control of learned vocalizations of songbirds, displays predictive activity that may enable the male to adjust his own calling pattern to produce very fast sequences of male female call exchanges.

## Introduction

Many group-living species, such as zebra finches, use a large numbers of calls to interact within their social group and, in particular, with their mates (Zann, 1996; Marler, 2004; Beckers and Gahr, 2010; Gill et al., 2015; Elie and Theunissen, 2016). During social interactions, each individual is confronted with mixtures of sounds that are emitted by various group members. Relevant information can only be retrieved and delivered with an effective system to process auditory inputs while controlling vocal outputs (Prather et al., 2008; Beckers and Gahr, 2012). In zebra finches, calling interaction patterns show that vocal responses can be fast (∼200 ms) and that calling becomes mate-specific during the breeding cycle (ter Maat et al., 2014; Gill et al., 2015). Previous computational and behavioral studies have shown that animals and humans benefit from predictive control that compensates for sensorimotor delays, thereby enabling effective sensory processing and motor response (Wolpert and Ghahramani, 2000; Wolpert et al., 2011). The ability to predict upcoming calling events may allow zebra finches to achieve such rapid and specific responses. While mechanisms of prediction have been studied in particularly in humans using fMRI techniques within the framework of decision neuroscience (Soon et al., 2008), little attention has been paid to the socio-sexual context and related selection pressures (Mobbs et al., 2018). Thus, the predictive control of social communication and the related neural mechanisms of prediction in sensorimotor systems are unknown.

To study the neural mechanisms of stimulus-response (sensorimotor) prediction underlying vocal interactions in a social context, we employed telemetric devices that enabled the continuous synchronized monitoring of vocal activity and of neurophysiological activity in HVC (proper name), a sensorimotor cortex-like nucleus known for its control of song pattern (Nottebohm and Arnold, 1976; Yu and Margoliash, 1996; Hahnloser et al., 2002). HVC is an important region of the song control system of zebra finches that has been used extensively to study vocal learning and the neural coding of birdsong (Doupe and Kuhl, 1999; Long and Fee, 2008; Mooney, 2009; Vallentin et al., 2016). With telemetric techniques, it has been shown previously that zebra finches respond more contingently to their sexual partners than to other group members (ter Maat et al., 2014; Gill et al., 2015) and that the premotor nucleus robust nucleus of the arcopallium (RA) plays a role in active calling in zebra finches (ter Maat et al., 2014). The motor cortex-like RA is the target area of HVC in the descending song control pathway of songbirds (Nottebohm and Arnold, 1976; Bolhuis and Gahr, 2006). HVC has a number of features that make it possible to predict the calling of a social partner to optimize vocal communication: (1) lesion of the HVC to RA connection disrupts the timing of call responses in playback experiments (Benichov et al., 2016); (2) the sequential timing of motor units is the function of HVC during singing (Yu and Margoliash, 1996; Hahnloser et al., 2002; Long and Fee, 2008); and (3) HVC obtains environmental information via a direct thalamic input (Wild, 1994; Akutagawa and Konishi, 2005; Coleman et al., 2007). In this study, we analyzed auditory and motor-related neuronal activity and their dependence on social context in the male HVC during calling interactions of freely behaving zebra finches.

## Materials and Methods

### 

#### Animals

Animal experiments were conducted according to the regulations of the government of Upper Bavaria (Az. 55.2-1-54-231-25-09). Experimental zebra finches were obtained from our breeding facility. Male and female zebra finches (>100 d post hatch) were used for the audiotelemetry and neurotelemetry experiments. Zebra finch pairs were kept in custom-made, sound-attenuated chambers. Each sound-attenuated chamber was equipped with a microphone (TC20, Earthworks), a speaker (FRS 8, 30 w, 8 Ω, VISATON) and a telescopic antenna for wireless recordings. Zebra finches were kept in a 14/10 h light/dark cycle (fluorescent lamps), 24°C, and 60–70% humidity.

#### Sound recording

Custom-made wireless microphones (0.6 g, including battery) were used for wireless sound recording (ter Maat et al., 2014). The wireless microphone was placed on the back and fixed with an elastic band around the upper thighs of the bird. The frequency modulated radio signals were received with communication receivers (AOR5000, AOR, Ltd.). Audio signals were either fed into an eight channels audio A/D converter (Fast Track Ultra 8R, Avid Technology) and recorded with custom-made software or registered on a DASH8X data recorder (Astro-Med).

#### Playback experiment

Playback experiments were performed in cohabitation (male and female were together in a same box) and in isolation (male was separated from female). Recorded stack calls of the female mates were played back by a speaker (FRS 8, 30 w, 8 Ω, VISATON). Female stack calls used for playbacks were recorded by the in-box microphone (TC20, Earthworks) before the experiments. The intervals between played-back calls were uniformly randomized between 1 and 30 s.

#### Implantation of electrode and chronic recording of neuronal activity

Male zebra finches that showed stable antiphonal interactions with their mates were chosen to carry the neural telemetric device. They were anesthetized using isoflurane inhalation (0.8–1.8% at 0.5l O_2_/min). During anesthesia, the animals were kept warm on a constant temperature pad (160 × 160 mm, 12 V/6 W, Thermo, GmbH) and wrapped in a thin gauze blanket. Some feathers of the head were removed, the skin was disinfected, opened and treated with lidocaine (Xylocain Gel 2%, AstraZeneca). After a window on the skull was opened, the bifurcation of the mid-sagittal sinus served as a reference coordinate. A 2-MΩ tungsten electrode (FHC) was lowered into HVC using a micromanipulator (Luigs and Neumann). After verifying the signals of HVC by playing back birds-own-song (BOS), conspecific songs and noise (Margoliash and Fortune, 1992), the window on the skull and the pins for both reference and recording electrode was covered and fixed with dental cement (Tetric evoflow, Ivoclar Vivadent, GmbH). The animal was equipped with a custom-made neural telemetric device and placed back into the sound box for chronic recordings of both vocal and neural signals (ter Maat et al., 2014). We monitored the neuronal activity during chronic recording. To ensure that the recording site was in HVC, we only selected recording sites that showed premotor activity in relation to singing, since several classes of HVC neurons including RA-projecting and X-projecting neurons and interneurons are active during singing (Kozhevnikov and Fee, 2007). For each recording site we found one stack call-related unit, while other units showed activity related to songs, distance calls or showed no specific activity. To further verify electrode placement, a lesion was made at the recording site by connecting the reference and the recording electrodes to the linear stimulus isolator (WPI, Inc). A current of 4.5 µA was applied for 6 s (ter Maat et al., 2014). The brains were then transferred to 4% paraformaldehyde and cryocut in 30-µm sections for Nissl staining. The lesion sites were confirmed with a standard light microscope (Leica, DM 6000B).

#### Amplitude of the stack calls and sensitivity of the backpack microphone

The sounds of males and females were recorded simultaneously with a calibrated central microphone (TC20, Earthworks) and with one backpack microphone mounted on the male and another backpack microphone mounted on the female. All events recorded by the central microphone during a 4-h period were compared with those recorded by the backpacks at that time. In all cases, the backpack microphones mounted on the female or male, respectively, recorded the calls emitted by the microphone carriers. The sound levels of calls were estimated by calibrating between the central microphone and the sound meter (HD600, Extech, with A-weighting, 125-ms response time, 1.4-dB accuracy).

#### Behavioral data acquisition and analyses

Vocal data were recorded continuously at 22,050-Hz sampling rate and written to WAV files. Vocalizations were extracted from audio files by using custom-written software (sound explorer). The vocalizations of zebra finches were clustered by analyzing their sound features (ter Maat et al., 2014). The peristimulus time histograms (PSTHs) indicate the times at which males called in relation to the onset of female calls (bin width: 50 ms). Z-scores were calculated as: $\frac{x-\mu }{\sigma }$, where *x*, μ, and σ are call counts, mean, and standard deviation (SD) of call counts, respectively.

#### Neurotelemetric recordings and analyses

The neurotelemetric signals were received and amplified with communication receivers (AOR5000, AOR, Ltd.). The signals were digitized (Fast Track Ultra 8R, Avid Technology) and recorded with custom-made software or registered on a DASH8X data recorder (Astro-Med). We used the same sampling rate (22,050 Hz) to record the neurophysiological data simultaneously with vocalizations of female and male zebra finches. Spike sorting was conducted offline by using Spike2 (CED). A template matching algorithm was applied for spike sorting in 300- to 10,000-Hz bandpass filtered recordings. Principal component analysis (PCA; Spike2) confirmed isolated units, and we obtained two to three units from each animal (eight birds). We selected one single-unit for each bird for further analysis that showed premotor activity associated with call production. The raster plots and the PSTHs indicate the times at which HVC neurons fired in relation to the onset of vocal events (bin width: 10 ms). Z-scores were calculated as: $\frac{x-\mu }{\sigma }$, where *x*, μ, and σ are spike counts, mean, and SD of spike counts, respectively. The Z-score is used for indicating a significant increase or decrease in activity with the critical value ±1.645 (α = 0.05). The signal-to-noise ratio (SNR) was calculated as: $10\times \log_{10}\frac{\sigma_{signal}^{2}}{\sigma_{noise}^{2}}$, where $\sigma_{signal}^{2}$ and $\sigma_{noise}^{2}$ are the variabilities of the signal and the noise, respectively. We used an *F* test to compare the neuronal activity patterns. We compared the neuronal activity in relation to associated female calls during cohabitation (coh-aF) against spontaneous female calls during cohabitation (coh-sF), associated female calls during separation (sep-aF), and spontaneous female calls during separation (sep-sF). In the context of separation, we separated female and male mates into two distant boxes with acoustic interconnections. The sum of the squared distances between the mean curves of coh-aF and individual curves of respective groups were calculated separately. The null hypothesis is that the difference (D_0_) between the mean curves of coh-aF and the individual curves of coh-aF yields the same variability as the difference (D_test_) between the mean curves of coh-aF and the individual curves of coh-sF, sep-aF, and sep-sF, respectively. The F-ratio between the sum of squared differences between D_0_ and D_test_ and the sum of squared difference within D_0_ and D_test_ was calculated. The degrees of freedom for between-groups and within-groups are *df_b_* and *df_w_*, respectively. The *p* value for the test is given by: $1-f_{cdf}(x,df_{b},df_{w})$, where *x* denotes F-ratio.

#### False discovery rate statistics of auditory-evoked HVC activity

In Figure 3, we analyzed eight single-units from eight male zebra finches for hearing-related activity in HVC. Of each unit, the spikes within [−1, −0.5), [−0.5, 0), [0, +0.5), and [+0.5, 1] s bins were counted in relation to the onsets of the female's stack calls, which were aligned at 0 s. For each neuron, we calculated the percentage of spikes occurring within the time window [−0.5, 0) s in relation to all spikes within the time window [−1, 1] s. Then we sorted the calling events according to the percentage of spikes occurring during [−0.5, 0) into 10 quantiles, 0–10% spikes, 10–20% spikes, and so on. Of the events of each quantile, we calculated the PSTHs (bin width: 10 ms) for the [−1, +1] s time window. For these PSTHs we calculated the Z-scores and defined the peak value of auditory-evoked HVC activity as the maximal Z-score within the time window [0, +0.5) s after the onset of female' stack calls (max Z-score_post_). The maximal Z-score within the time window [−0.5, 0) s preceding the female calls is defined as max Z-score_pre_. The Z-scores were converted to *p* values (*n* = 80, 10 quantiles of 8 birds). We controlled for the false discovery rate by using Benjamini–Hochberg procedure of *q* = 0.01 (Benjamini and Hochberg, 1995).

#### Experimental design and statistical analysis

Eight male and eight female zebra finches were used for the audiotelemetry and neurotelemetry experiments (cohabitation, separation). Three of these pairs were additionally used for the isolation experiment. Additional seven pairs were used for the behavioral experiment of call reaction times (RT) between mates during cohabitation and separation. In the context of cohabitation, zebra finch pairs were kept in custom-made, sound-attenuated chambers (cohabitation). In the context of separation, we separated female and male mates into two distant boxes with acoustic interconnections (the microphone and the speaker were interconnected). In the context of isolation, male zebra finches were kept alone in sound-attenuated chambers. Statistical analyses for behavioral and neurophysiological data were conducted using R (version 3.5.2). The behavioral data of RT were analyzed with paired *t* tests. We used *F* tests to compare the neuronal activity in relation to different contexts (cohabitation, separation, and isolation). False discovery rate statistics (p.adjust, R version 3.5.2) was also used to determine the dependency between the auditory-evoked activity and the predictive activity in HVC.

## Results

### Call-based communication between pair-bonded zebra finches

**Figure 1.**
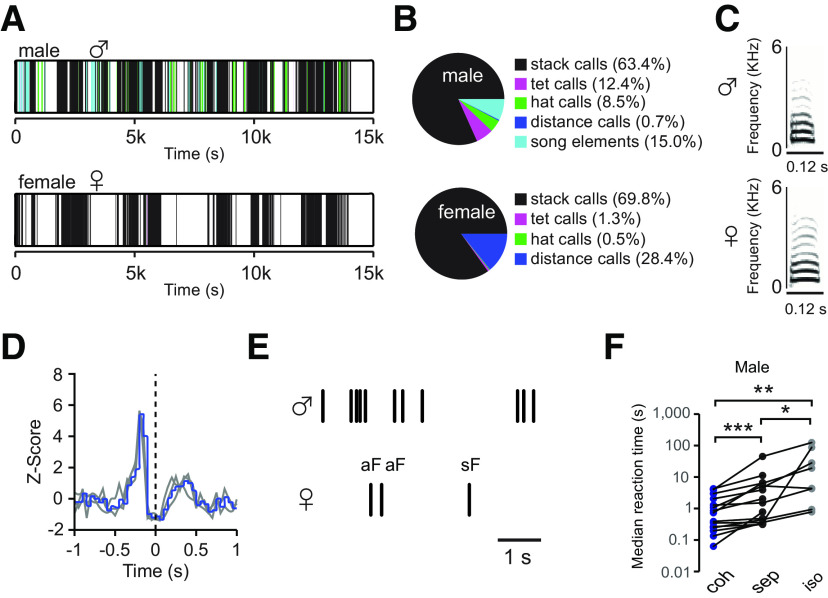
Vocal interactions between the male and the female of zebra finch pairs. ***A***, Successions of male and female vocal events of one pair. Each vertical line corresponds to a vocal event, and the color of the line relates to the vocal type shown in the pie chart. ***B***, The latter gives the proportions of different types of vocalizations in males and females; females lack song elements. ***C***, The sonograms show the stack call of a male and a female zebra finch. ***D***, The Z-score of male stack calls before and after female stack calls are plotted as a function of time with 50-ms time intervals; the onset of female stack calls was aligned at zero. Gray lines, Z-scores of the stack call interactions at three different (entire) days after pair bonding of a representative pair. In this case, the female responded to the male calls more frequently than the male responded to the female calls. Blue line, Average Z-score of 3 d. ***E***, Schematic illustration of an associated female call (aF) and a spontaneous female call (sF) in relation to the timing of male calls (black vertical lines). ***F***, Comparisons of the median reaction time (RT) of males in response to the female calls in different contexts: cohabitation (coh) = mate present; separation (sep) = mate can be heard but not seen; isolation (iso) = playback of the mate's calls. Significance index: ***p < 0.001, **0.001 < *p* ≤ 0.01, *0.01 < *p* ≤ 0.05.

Zebra finches live in loose social groups. Although all group members exchange various vocalizations, males and females address their calls with increasing selectivity to their mate during the progressing breeding cycle (Gill et al., 2015). Here, we study the dynamics of such dyadic vocal communications. Vocalizations of males and females were recorded simultaneously using telemetric microphones mounted on their backs, as described previously (Gill et al., 2015, 2016). While song is only uttered by males, male and female zebra finches share an extensive repertoire of calls (Fig. 1*A–C*), of which the stack calls were the most commonly uttered (65.5 ± 27.4% in male and 89.9 ± 4.8% in female; mean ± SD, 16 males, 16 females). Since stack calling is the preferred mode of communication of paired zebra finches, we chose stack calling for further analysis of neural mechanisms of natural vocal communications. Although the dynamic range of calling reactions in zebra finches is very large, their stack call communication exhibits an antiphonal pattern (ter Maat et al., 2014; Benichov et al., 2016; Ma et al., 2017; Pika et al., 2018). The temporal association of stack calls between male and female of zebra finch pairs showed the calling interaction pattern was consistent over days after pair bonding (Fig. 1*D*). Certain stack calls are temporally associated with the calling behaviors of their mates, while other stack calls are uttered spontaneously both without following and being followed by other calls. Forsimplicity, we indexed the former as associated calls (aF, associated female calls) and the latter as spontaneous calls (sF, spontaneous female calls), when the spontaneous calls occurred both without following and being followed by other vocalizations (including self-produced and other-produced calls and song elements) for half a second (Fig. 1*E*, aF is defined as the opposite).

To test the impact of social context on the stack calling dynamic, we separated male and female mates into two distant boxes with acoustic interconnections and compared the RT between the contexts of cohabitation and separation in male zebra finches. Because the distribution of the calling intervals in zebra finches is scale free (Ma et al., 2017) and only has finite weighted mean, we analyzed the medians of all intervals. Males increased their reaction time (RT) when answering their mates' stack calls during visual separation [sep; Fig. 1*F*, median RT_sep_ = 1.20 s (median 20% fastest RT_sep_ = 0.23 s); *p* < 0.001, paired *t* test, *n* = 15] and isolation [iso; Fig. 1*F*, median RT_iso_ = 2.61 s (median 20% fastest RT_iso_ = 0.43 s); *p* = 0.008, paired *t* test, *n* = 8] as compared with cohabitation [coh, median RT_coh_ = 0.94 s (median 20% fastest RT_coh_ = 0.15 s)], while the RT during isolation were longer than the RT during separation (Fig. 1*F*, *p* = 0.02, paired *t* test, *n* = 8).

### Telemetric monitoring of neuronal activity in male HVC during social interactions

**Figure 2.**
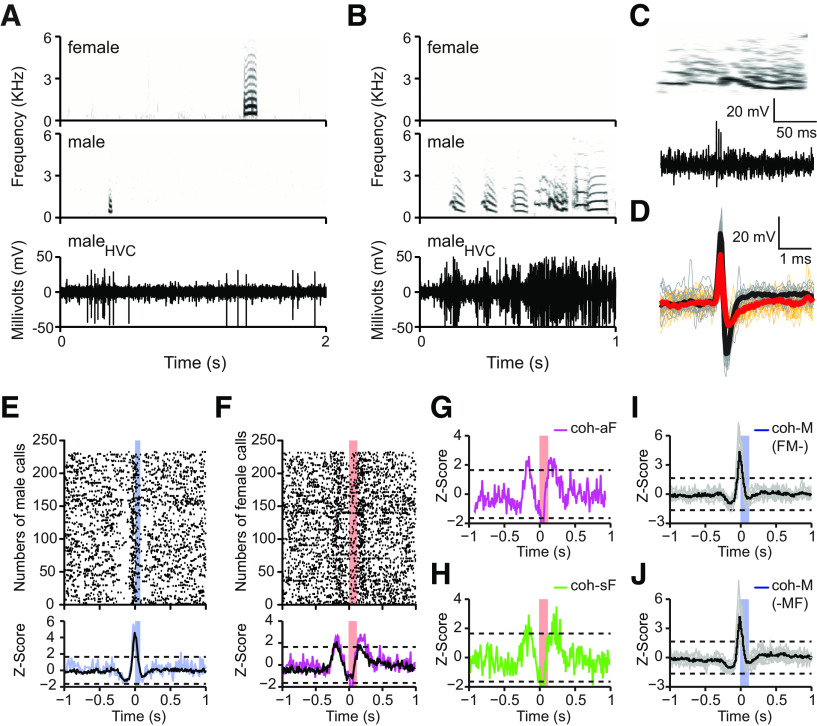
Neurotelemetry revealed both stack calling-related and hearing-related activity of the same HVC single-unit of male zebra finches during call-based vocal communications in the social context. ***A***, Raw extracellular voltage trace (lower panel) showing the HVC activity of one male in relation to the call of his female mate (upper panel) and to his own calling (middle panel). ***B***, Raw extracellular voltage trace showing the HVC activity of the stack call-related neuron of ***A*** in relation to singing. ***C***, Raw extracellular voltage trace (lower panel) showing the activity of a bursting unit during a song syllable (upper panels). ***D***, Comparison of the waveforms between stack call-related unit (black) and busting unit (red) recorded from the same animal. ***E***, PSTH of a stack call-related HVC single-unit with premotor activity in relation to the male's own active calling (stack calls, *n* = 233). Blue trace relates to the Z-score of one male; black trace to average Z-score of eight males. ***F***, Activity of the same HVC single-unit shown in ***E*** in relation to hearing of his female's stack calls (*n* = 233 randomly selected from 8689 female stack calls). Note that this unit shows a transient increase in activity already before the onset of the female calls (named predictive activity) and a second increase in activity following her calls (named auditory-evoked activity). Magenta trace relates to the Z-score of one male; black trace to average Z-score of eight males. ***G***, The activity pattern of the HVC unit shown in ***E*** in relation to the associated female stack calls (coh-aF) during cohabitation. ***H***, Activity of the same HVC unit shown in ***E*** associated with the spontaneous female stack calls (coh-sF) during cohabitation. Note that the putative predictive activity preceding the female call remained. ***I***, The activity pattern of male HVC units related to the onset of the males' calls in cases that the female called within the 500-ms time window before the males' call production (FM-). These female calls did not evoke auditory activity. ***J***, The activity pattern of male HVC units related to the onset of the males' calls in cases that the female called within the 500-ms time window after the males' call production (-MF). These females' calls did not evoke auditory activity. Gray and black traces represent the individual and the average Z-scores of eight males, respectively. Horizontal dashed lines indicate the significant Z-score = ±1.645. Blue and red color bars indicate the duration of male and female calls, respectively. The SNR of this single-unit is 3.8 dB.

We recorded the neuronal activity of the male HVC (*n* = 8 birds) with wireless telemetric device simultaneously with his own vocalizations and with those of his female mate. We show example of neurotelemetric recordings from the HVC of one male zebra finch during male-female vocal interactions (Fig. 2). HVC increased extracellular activity both in relation to calling and singing (Fig. 2*A*,*B*). We obtained two to three HVC single-units from each bird including one single-unit per recording site that showed premotor activity associated with productions of stack calls. We also recorded bursting activity associated with one specific type of syllables in the same animal (Fig. 2*C*). The stack call-related HVC units (Fig. 2*D*, black trace) recovered faster than did a bursting unit that did not show stack call-related activity (Fig. 2*D*, red trace). The waveform widths of stack call-related units at 25% peak amplitude were 0.22 ± 0.03 ms, which are comparable in spike width to those obtained with putative interneurons <0.3 ms (Rauske et al., 2003; Raksin et al., 2012). The previous studies and the spike waveform of the stack call-related HVC units suggest that these HVC units are putative interneurons (Kosche et al., 2015). The stack call-related HVC units exhibited premotor activity at −17.5 ± 13.9 ms before own calling. The stack call-related units had an average spontaneous firing rate of 7 ± 5 spikes/s. They became active during calling and singing and had firing rate of 116 ± 25 and 98 ± 28 spikes/s, respectively (*n* = 8 birds). In this study, we focused on stack call-related HVC units of males during female-male calling interactions.

In addition to what has been previously reported (McCasland, 1987; Margoliash, 1997; Hahnloser et al., 2002), we found that HVC activity occurred not only in relation to the males' own stack calls (Fig. 2*E*, the Z-score of one HVC single-unit in relation to onset of his calls) but also associated with the female's stack calls during call-based vocal interactions (Fig. 2*F*, same unit as in Fig. 2*E*; the Z-score of one HVC single-unit in relation to onset of female calls). The female call-related activity in male HVC included two peaks, one following the female's calls and one preceding the female's calls by ∼200 ms (Fig. 2*F*). The excitatory activity preceding the female calls was always followed by activity suppression that lasted ∼200 ms (Fig. 2*F*, the Z-score of one HVC single-unit in relation to numbers of female calls).

Since males produced different types of vocalizations (songs and various call types), including stack calls in association with the occurrence of the female stack calls, these associated calls could be the reason for the preceding activity, thus either being premotor or auditory evoked. If so, the spiking patterns of male HVC neurons related to spontaneous female calls (not associated with any other vocalizations including her own calls) should be different from the pattern related to the female calls associated with other calls of the male or female. Therefore, we compared the neuronal correlation of the associated female calls (coh-aF) with the neuronal correlation of the spontaneous female calls in the context of cohabitation (coh-sF). As similar as for the coh-aF (Fig. 2*G*, the Z-score of one HVC single-unit), both the HVC activity preceding and following the occurrence of the female's stack calls remained present for the coh-sF class (Fig. 2*H*, the Z-score of one HVC single-unit). Further, because of the definition of coh-sF calls, male calling was not related to these female calls.

It would have been possible that the coh-sF were associated with some very soft calls, which were not recorded by the backpack microphones or which were missed by the amplitude-based sound detection software. Although some female calls were at very low amplitude (27–31 dB), the backpack microphone recorded all sounds that were recorded by a calibrated standard microphone placed in the cage. Further, visual inspection of the sonograms of all coh-sF events confirmed the lack of low-amplitude female calls occurring briefly before the coh-sF. We were able to exclude the occurrence of very low-amplitude calls of the males uttered before the coh-sF since the backpack microphone recorded all calls.

During call production, HVC single-unit did not show auditory-evoked activity for female calls that occurred within 500-ms time window before (FM-) and after male calls (-MF; Fig. 2*I*,*J*, *n* = 8). Since the male HVC activity that preceded the female stack calling in the social setting was neither premotor nor auditory-evoked, we named this activity “predictive activity.” We discuss this term in relation to possible explanations such as repressed premotor activity in the discussion.

### In a social context, HVC neurons are auditory only if preceded by predictive activity

**Figure 3.**
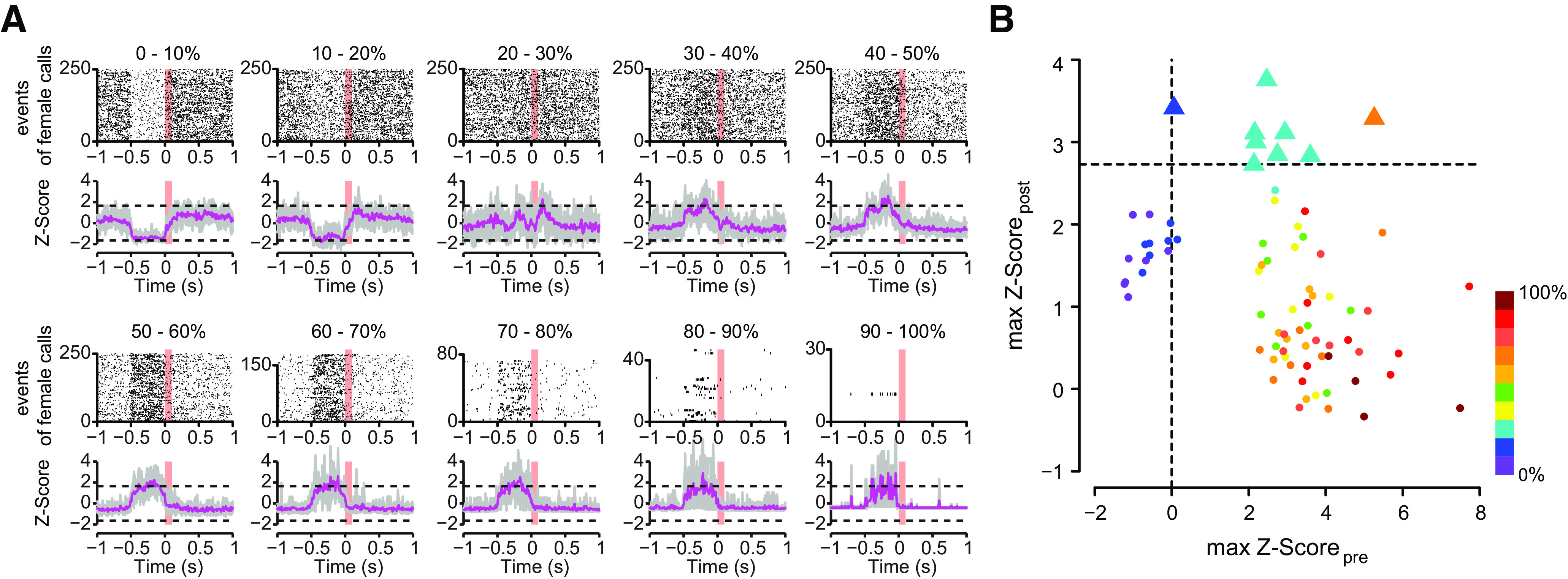
Hearing-related activity in male HVC according to different quantiles of spiking activity preceding the onset of female calls. ***A***, The upper and lower panels illustrate the raster plots of one bird and the average Z-scores of eight single-units (*n* = 8 birds), respectively. The dashed lines indicate the critical Z-score of ±1.645. Gray lines, The individual Z-scores of the single-units. Magenta lines, The average Z-scores of eight HVC single-units (*n* = 8 birds). Note that maximum of 250 events were randomly selected for raster plots. ***B***, The auditory-evoked HVC activity was significant (max Z-score_post_ > 2.73), if the activity preceding the females' calls increased (max Z-score_pre_ > 0). The maximum Z-score of HVC activity during 500 ms following the female call (max Z-score_post_) is shown as a function of the maximum Z-score of HVC activity during 500 ms preceding the female call (max Z-score_pre_). The quantiles are color coded according to the different quantiles of spiking activity preceding the onset of female calls (see Materials and Methods). The degree of HVC activity (0–100%) preceding the females' calls are shown from violet to dark red. Triangles indicate significant auditory-evoked activity with Z-score > 2.73 (corresponding to Benjamini–Hochberg false discovery rate of *q* = 0.01). Circles indicate events that were not significant; *n* = 8 HVC single-units sampled from eight male zebra finches.

We tested the relationship between the predictive activity and the auditory-evoked activity (see Materials and Methods, False discovery rate statistics of auditory-evoked HVC activity). We found significant auditory-evoked activity only in calling events, in which the activity increased transiently before the onset of the females' calls (Fig. 3*A*). This increased activity preceding the female calls corresponded to the predictive activity shown in Figure 2. We analyzed the correspondence between the auditory-evoked activity (max Z-score_post_) and the predictive activity (max Z-score_pre_) in relation to female calls. The female calls were sorted into 10 quantiles according to the percentage of spikes occurring half a second before the female calls (Fig. 3*B*, *n* = 8 birds). We only found nine (out of 80) cases with significant auditory-evoked activity (max Z-score_post_) after Benjamini–Hochberg corrections, in which seven cases of significant auditory-evoked activity were found to be contingent on there also being activity preceding the female calls (Fig. 3*A*,*B*, the 20–30% quantile, cyan triangle). These results suggest that, in the social context, a transient increase in predictive HVC activity is required for the auditory-evoked activity to occur in HVC.

**Figure 4.**
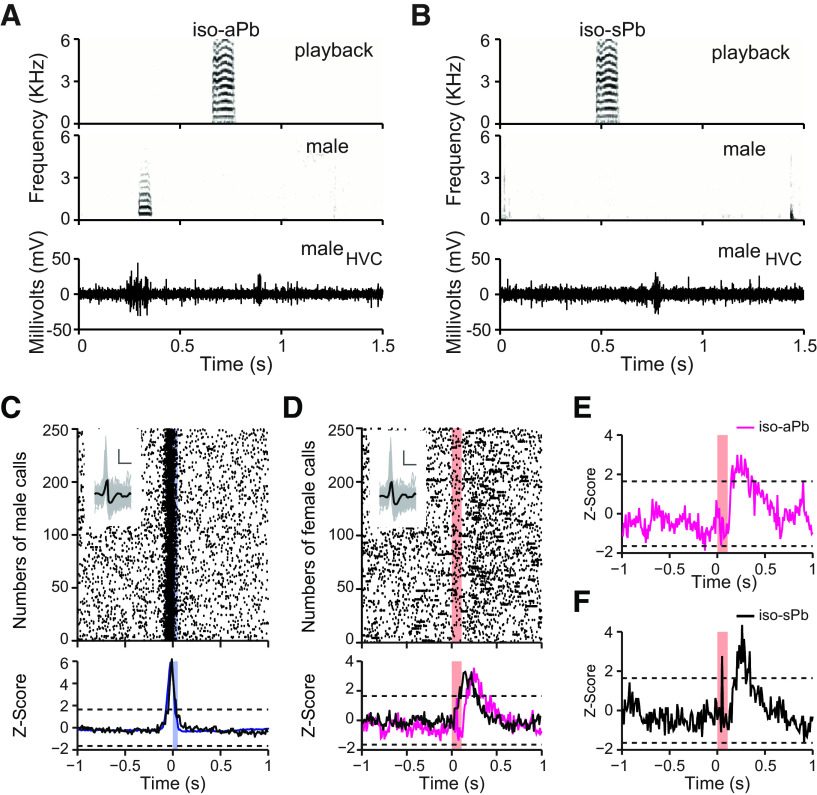
Calling-related premotor activity and auditory-evoked activity occurred in the same HVC single-unit in isolated males during playback experiments. In ***A***, the raw extracellular voltage trace (lower panel) shows the neural activity of a male in relation to the associated playback of his female's call (iso-aPb; upper panel) and to his own call (middle panel). In ***B***, the raw extracellular voltage trace (lower panel) shows the neural activity of the unit shown in ***A*** in relation to the spontaneous playback (iso-sPb) of his female's call (upper panel). This HVC unit fired stereotypically in relation to (***C***) active male calling (*n* = 250 calls randomly selected from 1629 own stack calls) and to (***D***) hearing of the played-back calls of his female mate (*n* = 250 playbacks randomly selected from 960 played-back female calls). The insert shows the spike shape of the depicted unit (vertical and horizontal scale bars: 50 mV and 0.5 ms). Blue and magenta traces represent the Z-score of one male in relation to call productions and hearing playbacks, respectively. Black trace represents the average Z-score of eight males. ***E***, ***F***, HVC unit's activity after splitting the playbacks into “associated” (iso-aPb) and spontaneous (iso-sPb) events during isolation. Note that auditory-evoked activity of HVC in the isolated male occurred without increased activity preceding the female calls. Blue and red color bars indicate the duration of male and female calls, respectively. The SNRs of this single-unit is 5.8 dB.

Next, we isolated the male from his female mate and played back her stack calls to him. The male HVC neurons showed the calling-related premotor activity in both isolated (Fig. 4*A*,*C*) and social context (Fig. 2*E*). In the isolated situation, the played-back calls would occasionally fall within the 500-ms criterion before and after a male call. We named these cases as iso-aPb (Fig. 4*A*). If the playback calls did not fall within 500-ms criterion, we named these cases as iso-sPb (Fig. 4*B*). In ∼60% of the playbacks (553 of 960 played-back calls), the male did not respond to the playbacks with calling and HVC neurons showed activity, which was auditory evoked (Fig. 4*B*,*D*). In this isolated context, there was no HVC activity preceding the hearing of the played-back female calls (Fig. 4*D*, the Z-score of one HVC single-unit in relation to numbers of played-back calls). This result remained even if we split the analysis of the playbacks into associated and “spontaneous” events; the auditory-evoked activity was significant in both cases [iso-aPb (Fig. 4*E*); iso-sPb (Fig. 4*F*)], while there was no predictive activity preceding the hearing of the playbacks. The average latency of the auditory-evoked activity (0.1 s, *n* = 3 birds) to the playbacks during isolation is shorter than the average latency (0.25 s, *n* = 8 birds) in relation to hearing of female calls in the social context (Mann–Whitney *U* test, *p* = 0.012).

### Male predicting requires context-dependent information from the female

**Figure 5.**
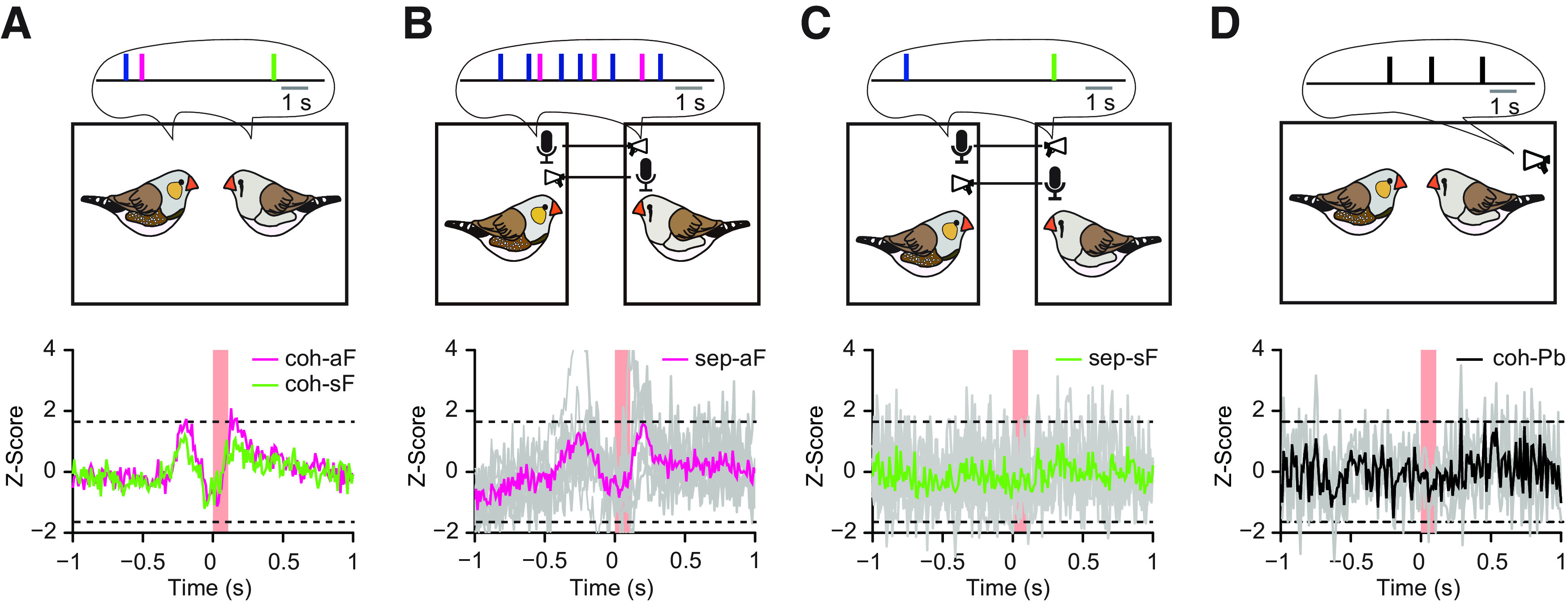
Neuronal activity of male HVC single-units in relation to female calls is context-dependent during calling interactions. ***A***, Comparing predictive and auditory-evoked activity of males' HVCs between the associated female calls (aF, magenta) and spontaneous female calls (sF, green) of their female mates in cohabitation (coh), *n* = 8 pairs. ***B***, Predictive and auditory-evoked activity in males' HVCs in relation to the aF in separation (sep), i.e., without visual input but with access to the females' vocal output, *n* = 6 pairs. ***C***, Lack of predictive and auditory-evoked activity in HVC in relation to the sF in separation (sep), where the females' vocal output during half a second preceding a focal call was eliminated, in addition to the missing visual input, *n* = 8 pairs. ***D***, Lack of auditory-evoked activity in the males in relation to the played-back calls of their female mates in cohabitation (coh), *n* = 3 pairs. Red bars, The durations of the females' calls and played-back females calls. Gray lines, The individual Z-scores of the single-units. Colored lines, The average Z-scores.

In order to predict upcoming calling events, male zebra finches must have acquired conjunctive information to foresee that their partners are about to vocalize. Conjunctive cues can either be the vocal context such as strings of related calls (Perez et al., 2015; Hernandez et al., 2016; vocal cues) or non-auditory social-sensory cues such as social gestures or postures (Partan and Marler, 2005), for simplicity referred to as visual cues. If neither vocal nor visual cues are available, the female call should not be predictable, and HVC should not be able to generate predictive activity related to upcoming female calls. In the cohabitation experiment (i.e., male and female being in direct contact), the predictive activity in response to the female calls was still detectable, although some of these female calls occurred spontaneously as in sF condition (Fig. 5*A*; see Fig. 2*F*, coh-sF vs coh-aF, *F*_(1,14)_ = 2.21, *p* = 0.16, *n* = 8 pairs). To test the importance of sensory cues further, we separated the female and male mates into two distant boxes with acoustic interconnections. Visual isolation alone had little impact on the predictive activity, which was similar to that of the cohabitation context (Fig. 5*B*; sep-aF vs coh-aF, *F*_(1,10)_ = 3.66, *p* = 0.08, *n* = 6 pairs). However, if the vocal cue was not available during visual isolation, when the female calls occurred unexpectedly without temporal association with either vocal or visual cues for half a second, the males no longer exhibited significant predictive activity or auditory-evoked activity in response to female calls (Fig. 5*C*; sep-sF vs coh-aF, *F*_(1,10)_ = 9.11, *p* = 0.01, *n* = 6 pairs). When we played back female calls while the male and the female were cohabitating in the same aviary, the male HVC did not show auditory-evoked activity in relation to the played-back female calls (Fig. 5*D*; coh-Pb vs coh-aF, *F*_(1,9)_ = 8.22, *p* = 0.02, *n* = 3). The playbacks were identical to those used in the isolated setting reported above (Fig. 4).

**Figure 6.**
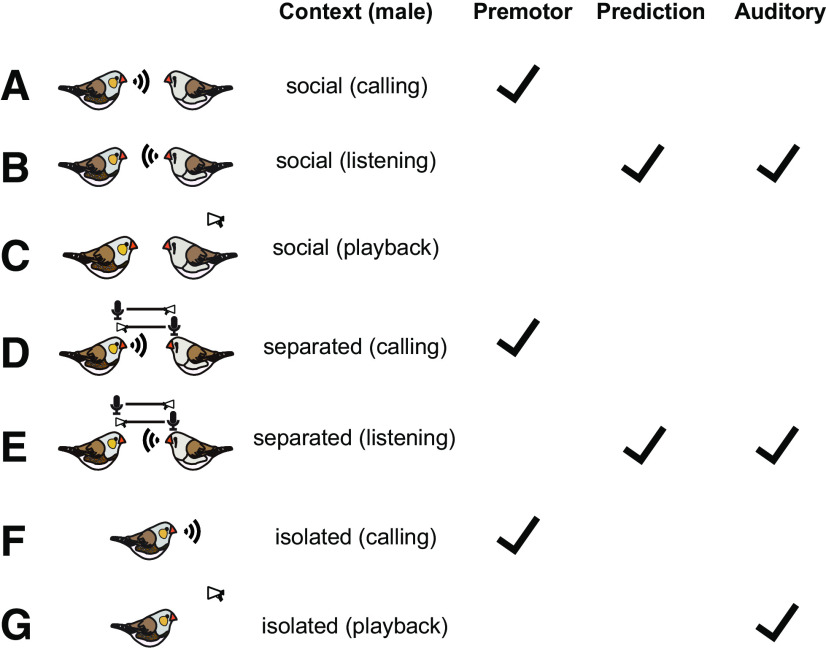
Summary of the task-dependent and social context-dependent activity in HVC neurons of male zebra finches. Stack call-related premotor activity occurred in each context (***A***, ***D***, ***F***). Auditory-evoked activity occurred in the social context only if that recording showed predictive activity (***B***, ***E***). The playbacks of female calls did not evoke auditory activity in male HVC during cohabitation (***C***). The playbacks of female calls evoked auditory activity in male HVC during isolation (***G***). Social: male and female cohabited in the same cage; separated: male and female had only auditory contact; isolated: male had no contact with the female.

In summary, for all studied males and HVC single-units, we found the following patterns of task-dependent and social context-dependent activity in HVC neurons of male zebra finches (Fig. 6): HVC single-units showed stack call-related premotor activity, regardless of whether the males were in social contexts, constrained social contexts (separation), or isolated contexts (Fig. 6*A*,*D*,*F*). In the social context, the HVC single-units showed predictive activity preceding the hearing of female calls in all males. The auditory-evoked activity occurred only subsequently to the predictive activity but not subsequently to premotor activity in the social context of all males (Fig. 6*B*). Despite constrained interactions in the context of separation, the HVC single-units displayed predictive activity and auditory-evoked activity (Fig. 6*E*). The playbacks of female calls did not evoke auditory activity in male HVC during cohabitation (Fig. 6*C*). When the playbacks became the only communication channel in the isolated context, HVC single-units displayed auditory-evoked activity in relation to hearing female calls but no predictive activity (Fig. 6*G*). Thus, in the social context, the males produce predictive activity that helps to gate auditory response that evokes HVC activity for calls of the social partner.

## Discussion

Zebra finches, like most other group-living songbirds, communicate using large numbers of acoustically invariant calls in addition to songs (Marler, 2004; Beckers and Gahr, 2010; Gill et al., 2015; Elie and Theunissen, 2016). Sensorimotor areas play a crucial role in motor planning (Riehle and Requin, 1989; Crammond and Kalaska, 2000; Maranesi et al., 2014), decision making (Wolpert et al., 2011), and the accuracy and timing of actions in response to sensory input (Wolpert et al., 1995; Hommel et al., 2013). The HVC of zebra finches is one of these sensorimotor areas, which is well known for its coding of temporal song patterns (Hahnloser et al., 2002; Long and Fee, 2008). Previous studies have occasionally reported calling related premotor activity in HVC neurons in the context of singing (McCasland, 1987; Margoliash, 1997; Hahnloser et al., 2002; Kozhevnikov and Fee, 2007). This calling-related activity in HVC was either pointed out in relation to the production of learned calls (Rajan, 2018) and was ignored in the more common unlearned calls (Hahnloser et al., 2002). Furthermore, bilateral damage of RA and HVC had no effect on the spectro-temporal morphology structure of innate calls of zebra finches (Simpson and Vicario, 1990), which was used to argue that HVC is not involved in the control of innate vocalizations of male and female songbirds. Since we detected neurons that participate in calling and auditory responses, we suggest that HVC is a general nucleus for vocal communication rather than a song-specific nucleus, likely responsible for both timing call production and predicted perception of calls, especially in a social context.

The stack call-related premotor activity of HVC interneurons is corroborated by recent results (Benichov and Vallentin, 2020). For singing, structured activity of interneurons is thought to provide permissive time windows for the activity of the descending premotor neurons, which leads to a sequential motor pattern (Kosche et al., 2015). During antiphonal callings, HVC interneurons might function in a similar way, that is, by pruning away a range of possibilities they determine narrow time windows in which call output in HVC is possible. Since individual interneurons were active in ∼90% of all stack calls, and since we found such neurons at each HVC recording site, we assume that most HVC interneurons are synchronously active at each call production. Thus, premotor activity likely involves both local and long-range excitatory and inhibitory circuits that lead to synchronized premotor activity of most HVC interneurons (Kosche et al., 2015; Kornfeld et al., 2017). Further studies should determine how the synchronized excitation of the HVC interneurons leads to the timed activity of RA-projecting HVC neurons, which affects the timing of antiphonal calling.

Instead of the premotor activity, the same neuron showed an increase of activity preceding female calls that was followed by an auditory-evoked activity in response to ∼20% of the female calls during antiphonal callings. This activity might be a predictive activity, generated to predict female calling or a fictive premotor activity of HVC interneurons that did not lead to a motor output but led to auditory-evoked responses. Considering that predictive/fictive activity does not occur in RA during stack call interactions (ter Maat et al., 2014), the predictive/fictive activity would happen in HVC. We suggest that the predictive/fictive activity is produced by a mechanism that reduces HVC-wide synchrony of interneurons involved in premotor activity, so that ∼10% of the interneurons are not active in timing call output. Those interneurons that do not participate in call timing might participate in premotor/fictive activity. Although interneurons can either be premotor or predictive/fictive during calling interaction, these two neuronal states do not occur within 500 ms from each other. Nevertheless, since most HVC interneurons contribute to the premotor activity during antiphonal callings, these neurons could determine the predictive/fictive activity that did not participate in the premotor activity, e.g., via HVC internal feedback circuits (Rosen and Mooney, 2006). However, since the predictive/fictive activity was generated even if the male did not call immediately before or after female calls (Fig. 2*H*), also other mechanisms could extract information of female's previous behaviors to generate predictive/fictive activity. In that case, the synchronized activity of a small fraction of HVC interneurons might not be sufficient to excite RA-projecting neurons, i.e., will not result in a motor output. The predictive/fictive activity might reflect the evolutionary history of HVC and the song control system in general as a network that evolved originally to control the timing of vocalizations such as innate calls. Only later during evolution, this auditory-vocal flexibility that originally concerned the time domain was extended to the spectral domain enabling vocal learning. Since the terms fictive or predictive matter only from an evolutionary perspective (Bubic et al., 2010) but not for the proximate effect of gated auditory responses, we continue the discussion using the term “predictive.”

The predictive premotor HVC activity was contingent on the activity of the same HVC interneuron activated by hearing the female call. During antiphonal callings, HVC interneurons seem to determine narrow time windows in which call processing in HVC is possible. Unlike motor performance, which can provide external feedback, the predictive activity may generate a feed-forward signal to auditory areas such as nucleus Avalanche [and caudomedial nidopallium (NCM) and caudal mesopallium (CM) interconnected with Avalanche] that respond to the incoming calls of the female mate, similar to an efferent copy of motor activity. Comparison of the timing of the male's predicting feed-forward signal, and of the timing of the female calls in the auditory areas, would result in an expectation error that measures the discrepancy between the predicted and the actual timing of female's calls. Neurons that detect errors in auditory feedback related to singing are present in CM (Keller and Hahnloser, 2009). Since Avalanche projects back to HVC (Akutagawa and Konishi, 2010; Roberts et al., 2017), Avalanche neurons would excite HVC in case of small expectation errors, which are calculated in Avalanche and related auditory areas. Large prediction errors would not excite HVC and might happen if the prediction was wrong, or if no prediction was computed; an example might be the lack of an auditory response to playbacks of female calls in the presence of a female since those playbacks occurred unexpectedly (Fig. 5*C*). We currently do not know whether Avalanche neurons excite HVC interneurons directly. In case of contingent excitation because of well-timed predictive activity and female calling, the excitatory input of Avalanche would result in auditory evoked activity in HVC interneurons. These complex computations would explain the delays of HVC activity when hearing female calls in a social context compared with the playback of her calls in a non-social context.

How might the perception of context affect HVC activity? The predictive HVC activity was still produced when we excluded all visual information or all short-term auditory information that preceded female calls. Since stack calling of zebra finch mates is steady (Ma et al., 2017) and follows a stable pair-specific calling pattern (Fig. 1*D*), the males seem to be able to extract information from the previous calling activity of females to predict future calling events during vocal interactions. Such auditory and visual information about the mate might reach HVC via Uva, which exerts synaptic influence on HVC interneurons and those projecting to RA and area X (Coleman et al., 2007). Uva obtains auditory input from the ventral nucleus of the lateral lemniscus (VNLL; Coleman et al., 2007), visual input from the optic tectum (Wild, 1994), and attentional input of the medial habenula (Akutagawa and Konishi, 2005). VNLL and Uva are, however, not sensitive to spectral details of sounds that would identify the calling individual (Coleman et al., 2007). This specific information could be provided to HVC by auditory association-cortex-like areas such as the NCM, CM, and nucleus Avalanche that are interconnected (Akutagawa and Konishi, 2010; Roberts et al., 2017) and are likely to analyze the auditory scene in a non-gated way (Beckers and Gahr, 2012; Elie and Theunissen, 2015). However, this auditory information is only reaching HVC in case of predictive activity in a social context or if the male is entirely in a non-social context where prediction is not an option. In relation to this, there are multiple mechanisms and pathways by which auditory responses in HVC can be gated (Cardin and Schmidt, 2004; Coleman et al., 2007).

An ability to predict allows animals to avoid surprise and to minimize energy cost in sensory sampling and processing (Friston, 2010). Alternatively, the ability to predict might enable the individual to signal fitness, i.e., to signal quality or commitment to a potential mate or to address a certain individual. Zebra finch pairs that are reproductively successful have more highly coordinated call interactions than those pairs that did not develop such cooperative calling behaviors (Gill et al., 2015). Birds may take advantage of expectations which optimize call timing to signal their own commitment for pair bonding or to evaluate the commitment of the female mate to the pair bonding.
